# Capsule Protects *Acinetobacter baumannii* From Inter-Bacterial Competition Mediated by CdiA Toxin

**DOI:** 10.3389/fmicb.2020.01493

**Published:** 2020-07-17

**Authors:** Renatas Krasauskas, Jūratė Skerniškytė, Julius Martinkus, Julija Armalytė, Edita Sužiedėlienė

**Affiliations:** Institute of Biosciences, Life Sciences Center, Vilnius University, Vilnius, Lithuania

**Keywords:** *Acinetobacter baumannii*, CdiA, capsule, BfmRS, contact-dependent inhibition

## Abstract

Currently, *Acinetobacter baumannii* is considered as one of the most important infectious agents causing hospital acquired infections worldwide. It has been observed that many clinically important pathogens express contact-dependent growth inhibition (CDI) phenomenon, which modulates cell–cell and cell–environment interactions, potentially allowing bacteria to adapt to ever-changing conditions. Mainly, these systems are used for the inhibition of the growth of genetically different individuals within the same species. In this work, by performing cell competition assays with three genotypically different (as determined by pulse-field gel electrophoresis) clinical *A. baumannii* isolates II-c, II-a, and II-a1, we show that *A. baumannii* capsule is the main feature protecting from CDI-mediated inhibition. We also observed that for one clinical isolate, the two-component BfmRS system, contributed to the resistance against CDI-mediated inhibition. Moreover, we were able to demonstrate, that the effector protein CdiA is released into the growth media and exhibits its inhibitory activity without the requirement of a cell–cell contact. Lastly, by evaluating the remaining number of the cells pre-mixed with the CdiA and performing live/dead assay, we demonstrate that purified CdiA protein causes a rapid cell growth arrest. Our results indicate, that capsule efficiently protects *A. baumannii* from a CDI-mediated inhibition by a clinical *A. baumannii* V15 strain, which is able to secrete CdiA effector into the growth media and cause target cell growth arrest without a cell–cell contact.

## Introduction

*Acinetobacter baumannii* is a Gram-negative coccobacilli, that has recently emerged as an important nosocomial pathogen (Peleg et al., 2008). The success of *A. baumannii* comes from the ability to display multi-drug resistance profile and persist in the surrounding environment (Harding et al., 2018). Currently, carbapenem-resistant strains are classified as the priority 1 pathogens by World Health Organization, indicating critical importance for the research and development of a new antibacterial strategies against *A. baumannii* (Tacconelli et al., 2018). Understanding how bacteria interact and respond to the environment is of great importance for the design of potential treatment strategies. One of the main mechanisms that allows *A. baumannii* to persist in the environment is through the use of various secretion systems (Weber et al., 2017; Harding et al., 2018).

Type V secretion systems are assigned into sub-types a–e, based on the export mechanism and general structure (Guérin et al., 2017). It has been determined that *A. baumannii* strains contain two Type V systems: Vb (AbFhaB/C and CdiA/B) and Vc (Ata) (Morris et al., 2019). Type Vb systems, also called two-partner secretion (TPS) pathway, consist of the secretory effector protein and its transporter across the outer membrane (Guérin et al., 2017). TPS systems have been shown to perform various functions such as secretion of virulence factors, adhesion, micro-nutrient uptake, and contact-dependent growth inhibition (CDI) within closely related strains (Willett et al., 2015b; Guérin et al., 2017).

Two-partner secretion systems that mediate CDI are seemingly widespread among Gram-negative bacteria (Aoki et al., 2010). They are composed of an outer membrane beta-barrel protein CdiB that allows the secretion of a large CdiA exoprotein onto the bacterial cell surface (Garcia, 2018). CdiA proteins in the most bacteria usually contain pretoxin-VENN motif, which marks the beginning of a highly variable C-terminal effector domain (CdiA-CT) of ∼200–300 amino acids. Some bacteria, such as *Burkholderia* sp., contain (Q/E)LYN motif. In the cases with no motif, CdiA-CT is identified as a high variability C-terminus region of CdiA (Willett et al., 2015b). CdiA-CT displays inhibitory activity and is translocated into the target cell. To evade the auto-inhibition by the CdiA toxin, bacteria encode a small immunity protein, which neutralizes the inhibitory activity of the CdiA-CT (Aoki et al., 2010; Garcia, 2018). The functional activity of the majority of CDI toxins can generally be classified into two types: the disruption of the target cell membrane by pore-forming activity or degradation/modification of cellular nucleic acids. Also, there have been observations that despite having a similar structure, toxins can have different substrate preferences (Willett et al., 2015b).

Only a few *Acinetobacter* Type Vb systems have been functionally characterized in depth. AbFhaB/FhaC system has been shown to facilitate the attachment to the human alveolar epithelial cells and contributes to the virulence against mice and nematodes (Pérez et al., 2017). Recently, this TPS was proposed to be a putative CDI system (Roussin et al., 2019). Interestingly, previous work failed to associate *Acinetobacter* CDI systems with the adhesion to surfaces, indicating that their main role is killing the neighboring genetically different individuals from the same species (Harding et al., 2017; De Gregorio et al., 2018; Krasauskas et al., 2019; Roussin et al., 2019).

A Recent *in sillico* analysis has identified that *Acinetobacter* sp. genomes encode up to two CDI systems, which can be separated into two classes by size and that there is a great heterogeneity within the sequences (De Gregorio et al., 2019). Our recent study shed some light on the regulatory mechanisms of these systems by showing that the two-component system BfmRS negatively regulates a CDI system from a clinical *A. baumannii* V15 strain (Krasauskas et al., 2019). In this study, by using genotypically different clinical *A. baumannii* strains with different surface related properties, we show that capsule is required for the protection against CDI-mediated activity. We also demonstrate, that two-component system BfmRS in some clinical isolates can mediate resistance to CDI. Finally, we show that the functional CdiA protein can be found secreted into the culture media and that the inter-cellular contact between aggressor and prey is not required for the CDI activity.

## Materials and Methods

### Bacterial Strains and Growth Conditions

*Escherichia coli* JM107 was used for all cloning experiments and DNA manipulations. *A. baumannii* and *E. coli* strains were grown in Luria-Bertani (LB) media or Tryptic soy broth (TSB) (Oxoid). All parent *A. baumannii* strains used in the work were previously characterized and are listed in Supplementary Table S1 (Povilonis et al., 2013; Krasauskas et al., 2019; Skerniškytė et al., 2019). The strains were classified according to Pasteur scheme for 7-gene multilocus sequence typing (Diancourt et al., 2010) and were determined to belong to sequence type (ST) 2 (*A. baumannii* II-c, *A. baumannii* II-a, and *A. baumannii* II-a1) and ST1422 for *A. baumannii* V15. Bacteria were grown at 37°C, unless indicated otherwise. Growth media were supplemented with antibiotics, where appropriate: ampicillin 100 μg/mL, gentamicin 10 μg/mL, and ceftazidime 10 μg/mL. Plasmids used in this study are listed in the Supplementary Table S1.

### Plasmid Construction

All reagents were obtained from Thermo Fisher Scientific. The procedures were performed according the manufacturer’s recommendations. Plasmids and primers used in the study are listed in the Supplementary Table S1, S2. All final constructs were verified by sequencing. Suicide plasmids for gene deletions were generated as described previously (Krasauskas et al., 2019). Complementation plasmids were constructed by firstly replacing the ampicillin resistance gene with the gentamicin resistance cassette *aac3I* in the leaky IPTG inducible shuttle expression plasmid from the previous study (Krasauskas et al., 2019). Then, the *gfp* gene in the newly created plasmid was replaced by the required gene amplified from the genomic DNA of the isolate of interest. The control plasmid pUC_AcORI_Ptac_TER_lacIq2_gm was obtained by removing the *gfp* gene and is denoted in figures as “p,” where relevant. The complementation plasmids containing the *cdiI*^V15^ and *bfmRS* genes were obtained from the previous study (Krasauskas et al., 2019), except *bla* gene in them was replaced with the *aac3I* cassette.

### Generation of *A. baumannii* Mutant Strains

A modified marker-less gene deletion technique from Oh et al. (2015) was used to obtain mutant strains of *A. baumannii* and is detailed in Krasauskas et al. (2019). Briefly, suicide plasmids, containing upstream and downstream regions of the genes to be deleted, were electroporated into the isolates of interest and selected on LB agar plates with 10 μg/mL of gentamicin. A single colony was inoculated into LB media without antibiotics and grown at 37°C for 4–6 h, after which the culture was streaked onto LB agar plates containing 10% sucrose and grown overnight at 37°C. Mutants were identified by PCR with specific primers (at least one of them was outside the region used for the suicide plasmid construction) and confirmed by sequencing. Primers that were used in the mutant construction are listed in the Supplementary Table S2.

### Fractionation of Capsular Polysaccharides

Capsular polysaccharide analysis was performed as described previously (Skerniškytė et al., 2019) with some modifications. Briefly, overnight cultures grown on LB agar plates were suspended in 1 mL of PBS to a final optical density (OD_600_) of 3. The suspensions were vigorously vortexed at a maximum speed for 120 s, followed by a centrifugation at 10,000 × g for 10 min. Capsular polysaccharides were precipitated by the addition of ethanol to a final concentration of 75% and centrifuged at 15,000 × *g* for 120 min at 4°C. The pellets were air-dried and re-suspended in Laemmli-SDS-PAGE sample buffer and boiled for 5 min. The samples were analyzed using the Laemmli-12% SDS-PAGE system. After electrophoresis, the gels were stained overnight with 0.1% (w/v) of Alcian Blue as described in Mercaldi et al. (2008).

### Inter-Bacterial Competition Assay

The assay was performed as described previously with some modifications (Krasauskas et al., 2019). Briefly, strains were grown overnight in TSB media at 37°C, washed twice with TSB, and diluted to a final concentration of 10^8^ colony forming units per milliliter (CFU/mL). The suspensions were mixed and competitions were performed at aggressor:prey ratio of 1:1 or 10:1 (indicated in figure legends). 5 μL of the resulting suspension was placed onto TSB media containing 1.5% agar and allowed to dry, followed by the incubation at 37°C for 6 h. The surviving bacteria were evaluated by plating serial dilutions of the re-suspended excised spot from the plate onto the LB agar plates containing gentamicin (10 μg/mL). All experiments included control reactions, which consisted of the non-aggressive *E. coli* strain DH5α mixed with each strain to obtain a total number bacteria if there was no competition between the strains. The obtained number of colonies were calculated as CFU per mL of culture.

### Biofilm Formation Assays

The biofilm formation assay was performed as described by O’Toole et al. (1999), with some modifications. Briefly, overnight cultures of *A. baumannii* strains grown in LB media at 37°C were diluted and inoculated into the wells of a round-bottom 96 well polystyrene microplates at a density of 10^7^ CFU/mL in LB. The cultures were incubated stationary at 37°C for 24 h under humid conditions. The formed biofilms were stained for 15 min with 0.1% (w/v) of crystal violet solution in deionized water. Prior to and after the staining, the wells were washed three times with deionized water. Biofilms were quantified by measuring OD_580_ of the dye that was solubilized with 33% glacial acetic acid for 15 min. The obtained values were normalized by a total bacterial biomass OD_600_. Experiments were performed three times with two technical replicates. Measurements were performed using Tecan Infinite M200 Pro plate reader.

### Protein Secretion Assay

The total protein content from culture media and cell fraction was assessed as described previously (Krasauskas et al., 2019) with some modifications. Briefly, strains were grown stationary in TSB media at 30°C for 30 h. The cells were separated from the media by a centrifugation at 10,000 × *g* for 10 min at 4°C. The supernatant was further clarified by a filtration through 0.22 μm filter to remove the remaining biomass. The proteins were precipitated by the addition of trichloroacetic acid (TCA) to a final concentration of 10% and centrifuged at 15,000 × *g* for 60 min at 4°C. The pellet was washed twice with an ice-cold acetone and dried by incubating tube at 95°C. The separated cells and precipitated proteins were then re-suspended with Laemmli-12% SDS-PAGE sample buffer. After electrophoresis, gels were stained with Coomassie brilliant blue. The PageRuler^TM^ unstained broad range protein ladder (5 μl per lane) was used as a marker (Thermo Fisher Scientific).

### *A. baumannii* Growth Assays

Clinical *A. baumannii* isolates and their derivatives were grown overnight in LB media at 37°C with shaking. The cultures were diluted to 10^7^ CFU/mL and 10-fold dilutions of the suspensions were then inoculated into a flat-bottom 96 well polystyrene microplate to a final volume of 150 μL LB. The cultures were grown until early logarithmic phase (OD_600_ of 0.25–0.35), after which the required additive was added in a total volume of 10 μL and the growth was allowed to continue. The monitoring was performed at 37°C using Tecan Infinite M200 Pro plate reader with shaking. Experiment was repeated three times with two technical replicates each. The following additives were used where necessary: 0.22 μm filtered supernatant from the growth media, sterile PBS buffer, sterile LB media, purified CdiA protein at 0.3 μg/mL final concentration, purified CdiA protein pre-incubated for 30 min at 37°C with Proteinase K (10 μg/mL) at a final concentration of 0.3 μg/mL.

### Purification of CdiA

CdiA was purified from the 750 mL supernatants of *A. baumannii* V15 Δ*bfmRS* cells that were grown stationary in TSB media for 30 h at 30°C. The cultures were then centrifuged at 10,000 × *g* for 10 min, followed by a filtration through 0.22 micron regenerated cellulose filter to remove the residual cells. Proteins in the supernatant were precipitated by a slow addition of ammonium sulfate under constant mixing to a final concentration of 40%. The precipitate was obtained by a centrifugation at 15,000 × *g* for 25 min. The pellet was re-suspended in 10 mL 0.05 M sodium phosphate buffer (pH 7.0), containing 10% ammonium sulfate. The CdiA protein was purified by the hydrophobic interaction chromatography (HIC), using 1 mL Butyl Sepharose 4 Fast Flow column (GE Healthcare) on the ÄKTA FPLC system (GE Healthcare). The column was equilibrated with 0.05 M sodium phosphate, 1.0 M ammonium sulfate, pH 7.0. After loading the protein precipitate, the column was washed with the same buffer for 5 column volumes followed by a linear gradient over 14 column volumes using 0.05 M sodium phosphate, pH 7.0 buffer. The fractions with the eluted CdiA were pooled and concentrated with a buffer exchange into PBS using Pierce^TM^ Protein Concentrator PES, with a 100 kDa cutoff value (Thermo Fisher Scientific). Protein concentration was determined using Roti^®^Quant Bradford protein assay (Roth) and bovine serum albumin as a calibrating protein.

### Protein Size Determination by Size-Exclusion Chromatography

The purified CdiA was analyzed by FPLC gel-filtration chromatography using Superose 12 10/300 GL column (GE Healthcare) on the ÄKTA FPLC system (GE Healthcare). The column was equilibrated with PBS. Gel filtration flow rate was set at 0.6 mL/min. The protein molecular mass standards (Amersham biosciences) were selected as follows: Blue Dextran, ferritin (440 kDa), catalase (232 kDa), aldolase (158 kDa), bovine serum albumin (68 kDa). To additionally confirm results, 0.5 mL fractions were collected, subjected to SDS-PAGE analysis and to grow inhibition assay as described above.

### Bacterial Viability Assay

Overnight cultures of bacteria grown in LB media were diluted in a microplate wells to a final concentration of 10^8^ CFU/mL in a volume of 100 μL. Next, 5 μL of either purified CdiA (5 μg/mL) or PBS as a control, were added to the suspensions. Mixes were incubated at 37°C and serial dilutions were plated at various time points onto LB agar plates to enumerate viable bacteria.

### Live/Dead Staining and Microscopy

For live/dead assay, overnight bacteria cultures were washed twice with a sterile PBS. Washed bacteria were diluted to a final concentration of 10^8^ CFU/mL in a total volume of 250 μL as follows: in PBS (control for live bacteria); 70% isopropanol (control for dead bacteria); PBS with CdiA (5 μg/mL final concentration). After incubation for 30 min and 3 h at room temperature, the cells were washed 3 times with PBS at 5,000 × *g* for 2 min, concentrated to a cell density of 10^9^ CFU/mL and subjected to cell staining for 15 min in the dark using SYTO9 (Invitrogen) and propidium iodide (Sigma) at a final concentration of 9.4 nM and 42.4 nM, respectively. 5 μL of cells were trapped between a slide and an 18 mm square cover-slip and visualized at 1,000× magnification with a fluorescence microscope Olympus AX70 equipped with 100×/1.35 oil immersion lens and WIBA (460–490 nm for excitation and 515–550 nm for emission) and MWG (510–550/590) filter cubes for SYTO9 and propidium iodide, respectively. The images were recorded with a CCD camera Orca (Hamamatsu). The images for SYTO9 and propidium iodide were taken under the same acquisition setting. Bright field was used to visualize all cells. The obtained images were merged into a single pseudo-color image using Fiji software (Schindelin et al., 2012). The surviving cells before and after each treatment, were washed and enumerated by plating on selective plates containing 10 μg/mL gentamicin to confirm the bacterial viability assay results.

### Statistical Analyses

All statistical comparisons were performed using one-way ANOVA (*p* = 0.05) with a Tukey HSD *post hoc* test. Inter-bacterial competition and viability assays were calculated as follows: CFU per mL was normalized by taking the decadic logarithm and using these values for statistical analysis. Asterisks in the figures denote the statistically significant difference between the groups (n.s., not significant; ^∗^*p* < 0.05; ^∗∗^*p* < 0.01; ^∗∗∗^*p* < 0.001). The analyses were performed using R package (version 3.2.3). Graphs were drawn using QtiPlot.

## Results

### Capsule Protects Target Cell From CDI-Mediated Attack

In the previous study, we identified that the two-component system BfmRS negatively regulates CDI system in *A. baumannii* V15 clinical strain. However, even using *A. baumannii* V15 Δ*bfmRS* strain with an up-regulated CDI as an aggressor in the competition assays against two clinical *A. baumannii* strains, we observed that only one of them was highly susceptible (Krasauskas et al., 2019). We hypothesized that the formation of capsule may play a protective role against CDI-mediated inhibition as this was one of the differences between the strains (Krasauskas et al., 2019; Skerniškytė et al., 2019).

To test this hypothesis, we selected three target *A. baumannii* clinical strains (II-c, II-a1, and II-a), belonging to distinct genotypically related groups – pulsotypes (evaluated by pulse-field gel electrophoresis) from the collection of clinical isolates obtained in Lithuanian tertiary care hospitals (Povilonis et al., 2013; Skerniškytė et al., 2019). These strains were determined to contain KL2 capsule locus type according to the amplification of the conserved *wzy* gene with KL2-specific primer pair (Skerniškytė et al., 2019). We also determined that although these strains belonged to sequence type 2 according to Pasteur classification scheme, and displayed similar capsular polysaccharide profiles, they differed between each other in the resistance to desiccation, hydrophobicity, ability to be phagocytosed by macrophages, and virulence against *Caenorhabditis elegans* (Skerniškytė et al., 2019).

**FIGURE 1:**
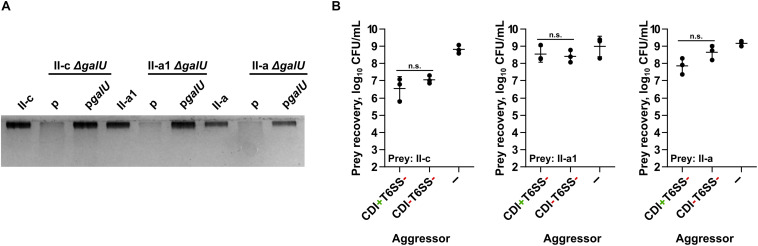
**(A)** Evaluation of capsular polysaccharide production by clinical strains II-c, II-a1, and II-a, their *galU* mutants, and *galU* mutants complemented with the wild-type *galU* gene. A part of 12.5% SDS-PAGE gel stained with Alcian Blue is shown. **(B)** Quantitative evaluation of inter-bacterial competition. Graphs show recovered numbers of *A. baumannii* clinical strains II-c, II-a1, and II-a after being premixed with an excess of an aggressor at the aggressor:prey ratio of 10:1. Competitions were performed with the following *A. baumannii* V15 mutants used as the aggressors: Δ*bfmRS*Δ*hcp* (CDI^+^T6SS^–^), Δ*bfmRS*Δ*hcp*Δ*cdi^V15^* (CDI^–^T6SS^–^). *E. coli* DH5α was used as a negative non-competitive control to enumerate bacteria numbers if there was no competition. Error bars represent standard deviation. Horizontal lines represent mean value. Values were calculated from three independent experiments. n.s., not significant.

In the selected strains via markerless deletion technique we introduced *galU* gene deletions. The *galU* gene encodes a predicted UTP-glucose-1-phosphate uridylyltransferase and its loss has been shown to result in the deficiency of capsular polysaccharides in *A. baumannii* (Geisinger and Isberg, 2015). The deletions were confirmed by sequencing. The loss of polysaccharide production in the mutants was verified by 12.5% Laemmli-SDS-PAGE followed by staining with alcian blue (Figure 1A). The polysaccharide production in *A. baumannii galU* mutants was readily restored by the introduction of a native *galU* gene, when supplemented in *trans* by the plasmid p*galU* (Figure 1A). Additionally, by PCR using primer pair Cdi_Imm_F/Cdi_Imm_R we confirmed that the selected *A. baumannii* target strains did not contain the same immunity gene to that of the aggressor strain (*A. baumannii* V15), indicating that they do not contain identical CDI system. Also, by performing competition assays using an excess of aggressor to prey ratio of 10:1, we confirmed that they are somewhat resistant to the CDI-mediated killing (Figure 1B).

We then performed competition assays selecting *A. baumannii* V15 as the aggressor strain and II-c, II-a1, II-a, and their derivatives as the target strains. It must be noted that we selected *A. baumannii* V15 Δ*bfmRS*Δ*hcp* mutant as the aggressor strain due to the fact that it not only displayed up-regulated *cdi* locus expression (compared to WT), but also had an inactivated Type VI secretion system (T6SS), in order to eliminate a potential T6SS influence on the competitions (Krasauskas et al., 2019). We denoted Δ*bfmRS*Δ*hcp* mutant as CDI^+^T6SS^–^ strain. To evaluate whether the inhibition was CDI-mediated, we also used Δ*bfmRS*Δ*hcp*Δ*cdi^V15^* mutant with no apparent inhibitory activity (denoted as CDI^–^T6SS^–^). The competitions were performed using the aggressor:prey ratio of 1:1 (based on CFU/mL) as the preliminary experiments showed a high sensitivity of mutants to CDI-mediated competition.

**FIGURE 2:**
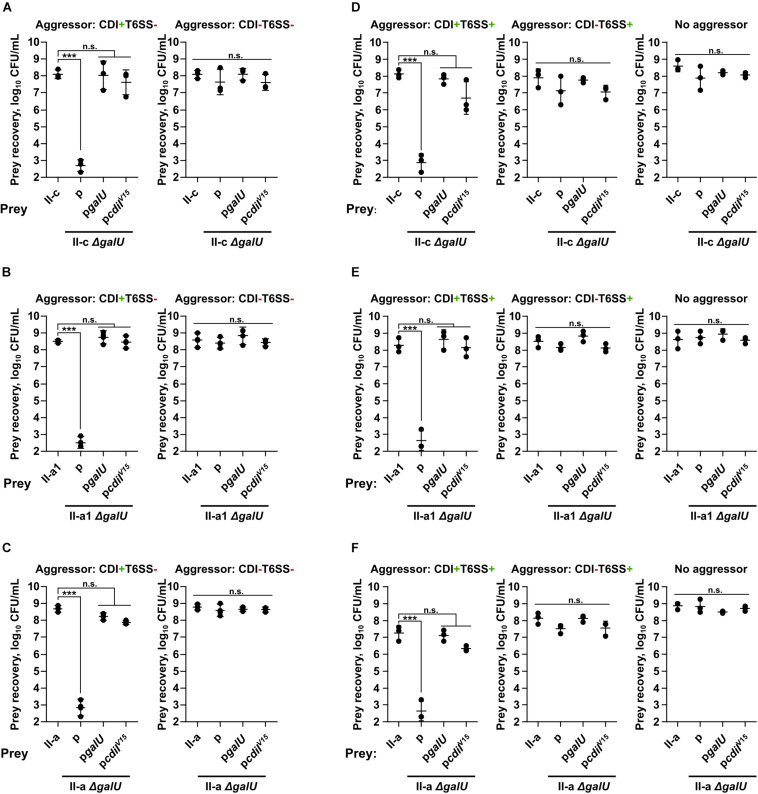
Quantitative evaluation of inter-bacterial competition. Graphs show recovered numbers of *A. baumannii* clinical strains after being premixed with an aggressor at the aggressor:prey ratio of 1:1. *A. baumannii* clinical strains II-c **(A,D)**, II-a1 **(B,E)**, and II-a **(C,F)**, their *galU* mutants, and *galU* mutants complemented with either wild-type *galU* gene or immunity *cdiI*^V15^ gene from the *A. baumannii* V15 strain were used as prey. Competitions were performed with the following *A. baumannii* V15 mutants used as the aggressors: Δ*bfmRS*Δ*hcp* (CDI^+^T6SS^–^) **(A–C)** Δ*bfmRS*Δ*hcp*Δ*cdi^V15^* (CDI^–^T6SS^–^) **(A–C)**, Δ*bfmRS* (CDI^+^T6SS^+^) **(D–F)**, Δ*bfmRS*Δ*cdi^V15^* (CDI^–^T6SS^+^) **(D–F)**. *E. coli* DH5α was used as a negative non-competitive control to enumerate bacteria numbers if there was no competition. Error bars represent standard deviation. Horizontal lines represent mean value. Values were calculated from three independent experiments. Statistically significant differences are indicated by three asterisks (One-way ANOVA, *p* < 0.001); n.s., not significant.

As can be seen in Figures 2A–C, the loss of capsule decreased the resistance of prey strains by ∼5–6-orders of magnitude, when they were attacked by the aggressor with an active CDI (CDI^+^T6SS^–^), but not when they were competed against the CDI-negative (CDI^–^T6SS^–^) strains. The susceptibility to CDI-mediated inhibition of all *A. baumannii* Δ*galU* strains was rescued by the complementation with the p*galU* plasmid encoding a native *galU* gene (Figures 2A–C). To additionally confirm, whether the observed inhibition was mediated via CDI mechanism, we also complemented prey Δ*galU* strains with the p*cdiI^V15^* plasmid, encoding immunity gene from the aggressor strain, and observed that prey strains were also rescued from the inhibition (Figures 2A–C).

Additionally, we performed competitions using aggressors that also displayed T6SS activity, namely the Δ*bfmRS* mutant, which had both CDI and T6SS activity (CDI^+^T6SS^+^), and the Δ*bfmRS*Δ*cdi^V15^* mutant with only T6SS activity (CDI^–^T6SS^+^). Results were consistent with the data above, as prey numbers were reduced when the competitions were performed using T6SS^+^CDI^+^ aggressor, while T6SS^+^CDI^–^ strain was unable to out-compete capsule negative mutants (Figures 2D–F). These results showed, that capsule deficient stains were killed via CDI system and that capsule was crucial for *A. baumannii* to evade CDI-mediated inhibition.

### BfmRS System in Some Clinical Strains Protects From CDI

Previously published data suggested that in some *A. baumannii* strains the loss of the two-component system BfmRS leads to the down-regulation of capsule locus and reduced capsule production (Geisinger and Isberg, 2015; Geisinger et al., 2018). Therefore, we ought to test the hypothesis, whether the loss of BfmRS system would lead to the increased susceptibility of clinical isolates to CDI-mediated inhibition.

**FIGURE 3:**
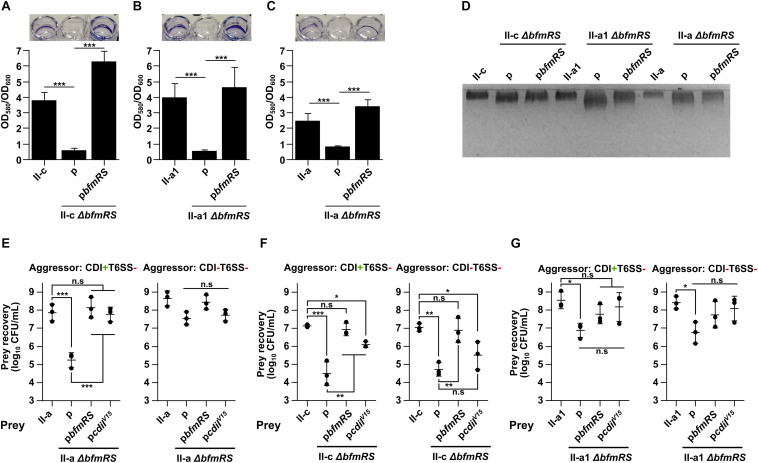
**(A–C)** Evaluation of biofilms formed by *A. baumannii* strains II-c **(A)**, II-a1 **(B)**, and II-a **(C)**, their *bfmRS* mutants, and *bfmRS* mutants complemented with the wild-type *bfmRS* operon from *A. baumannii* V15. Biofilms were quantified from at least three independent experiments performed in duplicates and normalized by dividing by the total growth (OD_600_). Pictures above graphs show macroscopic images of biofilms stained with crystal violet. **(D)** 12.5% SDS-PAGE gel stained with Alcian Blue showing capsular polysaccharide profiles of clinical strains I-c, II-a1, and II-a, their *bfmRS* mutants, and *bfmRS* mutants complemented with wild-type *bfmRS* operon. **(E–G)** Quantitative evaluation of inter-bacterial competition. Graphs show recovered numbers of *A. baumannii* clinical strains after being premixed with an aggressor at the aggressor:prey ratio of 1:1. *A. baumannii* clinical strains II-a **(E)**, II-c **(F)**, and II-a1 **(G)**, their *bfmRS* mutants, and *bfmRS* mutants complemented with either wild-type *bfmRS* operon from *A. baumannii* V15 or immunity gene *cdiI*^V15^ from the *A. baumannii* V15 strain were used as prey. Competitions were performed with the following *A. baumannii* V15 mutants used as aggressors: Δ*bfmRS*Δ*hcp* (CDI^+^T6SS^–^), Δ*bfmRS*Δ*hcp*Δ*cdi^V15^* (CDI^–^T6SS^–^). Error bars represent standard deviation. Horizontal lines represent mean value. Values were calculated from three independent experiments. Statistically significant differences are indicated by one asterisk (One-way ANOVA, *p* < 0.05), two asterisks (One-way ANOVA, *p* < 0.01), or three asterisks (One-way ANOVA, *p* < 0.001); n.s., not significant.

Firstly, we generated Δ*bfmRS* mutants in all three selected clinical isolates (II-c, II-a1, and II-a) and confirmed the deletions by sequencing and by performing biofilm formation assays, as the loss of BfmRS leads to the inhibition of biofilm formation (Tomaras et al., 2008). As can be seen in Figures 3A–C, the ability of the mutants to form biofilms on plastic was completely abolished unless they were complemented with the whole *bfmRS* operon *in trans*. Next, we evaluated the polysaccharide production of each of the mutants expecting to observe the reduced capsule production. Surprisingly, we did not observe apparent differences in the quantity of capsular polysaccharide production between WT strain and its respective Δ*bfmRS* mutant (Figure 3D). However, the Δ*bfmRS* mutants displayed a wider capsular polysaccharide profiles, compared to the parent strains, suggesting a possible modifications in the general structure of the capsule (Figure 3D).

We then performed competition assays to test whether the loss of BfmRS system would influence the susceptibility of clinical isolates to CDI-mediated inhibition. As an aggressor, we selected *A. baumanni*i V15 Δ*bfmRS*Δ*hcp* mutant (CDI^+^T6SS^–^). Intriguingly, we observed that the Δ*bfmRS* mutant of II-a strain, indeed became susceptible to CDI-mediated inhibition by the CDI^+^T6SS^–^ strain (Figure 3E). The resistant phenotype was fully reinstated, when the mutant was complemented with the p*bfmRS* or p*cdiI*^V15^ plasmids (Figure 3E). Consistently, when the aggressor strain was lacking a functional CDI system (CDI^–^T6SS^–^), the cell count of the Δ*bfmRS* mutant was similar to that of the WT strain or the complemented strains with the p*bfmRS* or p*cdiI*^V15^ plasmids (Figure 3E). It must be noted that the remaining Δ*bfmRS* strains, namely II-a1 and II-c behaved very differently. II-c Δ*bfmRS* mutant was readily susceptible to both CDI^+^T6SS^–^ and CDI^–^T6SS^–^ aggressors, possibly indicating a general growth deficiency in co-cultures (Figure 3F). And while it was partially rescued by the p*cdiI*^V15^ plasmid, when competed against CDI^+^T6SS^–^ (Figure 3F), due to the susceptibility to CDI^–^T6SS^–^, we could not conclude that the BfmRS system in this strain causes resistance to CDI-mediated inhibition. However, complementation with the p*bfmRS* plasmid reinstated the phenotype to the WT strain (Figure 3F), indicating, that the system may play a protective role during competition with other strains. Competitions using II-a1 Δ*bfmRS* mutant resulted in a reduced cell count. However, we could not conclude that the BfmRS system was responsible for the protective role in this strain as complementation with either the p*bfmRS* or p*cdiI*^V15^ plasmids did not significantly increase the cell count after the competition (Figure 3G).

Collectively, the results discussed above, show that at least in one clinical *A. baumannii* strain the BfmRS system is involved in the protection against CDI-mediated inhibition. The results also suggest, that the system might play a strain-specific role in *A. baumannii* and in different strains mediates different physiological outcomes in co-cultures.

### *A. baumannii* Secretes CdiA Protein and Does Not Require Cell–Cell Contact for CDI

**FIGURE 4:**
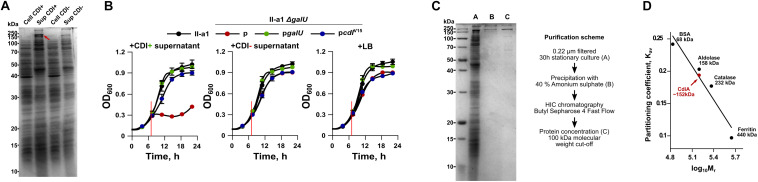
**(A)** An SDS-PAGE analysis of total protein fraction from cell extract (Cell) and culture media (Sup) of both *A. baumannii* V15 Δ*bfmRS* (CDI^+^) and Δ*bfmRS*Δ*cdi^V15^* (CDI^–^) strains. Proteins from culture media were precipitated with trichloroacetic acid (TCA). Samples were run on a 12.5% SDS-PAGE gel. Approximately 5 μg of the total protein were loaded into each well. The red arrow denotes CdiA protein band. **(B)** Growth inhibition assays of II-a1 strain, its *galU* mutant, and *galU* mutant complemented with either wild-type *galU* gene or immunity gene *cdiI*^V15^ from the *A. baumannii* V15 strain. Red vertical lines denote the time when 10 μl of 0.22 μm filtered culture media from Δ*bfmRS* (CDI^+^), Δ*bfmRS*Δ*cdi^V15^* (CDI^–^), or LB, were added. Error bars represent standard deviation. Values were calculated from three independent experiments with two technical replicates each. **(C)** 12.5% SDS-PAGE gel showing purity of CdiA after each purification step. The scheme of CdiA purification is denoted on the right. Approximately 10 μg of the total protein were loaded into the well with TCA-precipitated total protein fraction from culture media, and 1 μg of the total protein for all other wells. **(D)** Calibration curve used to determine the molecular weight of CdiA by size-exclusion chromatography. Black dots with names and their sizes (in kDa) denote proteins used for molecular mass standard curve. Red dot denotes calculated CdiA molecular mass. *K*_av_ denotes molecule partition coefficient, calculated as (*V*_e_ – *V*_o_)/(*V*_t_ – *V*_o_), where *V*_e_ – elution of the molecule (mL), *V*_o_ – column void volume (mL), and *V*_t_ – total column volume (24 mL). Log_10_M_r_ denotes decadic logarithm of the molecular weight. For SDS-PAGE analyses, PageRuler^TM^ Unstained Broad Range Protein Ladder (Thermo Fisher) was used as a marker. Numbers on the left of the gels denote molecular mass in kDa. Bands in gels were stained with Coomassie Brilliant Blue.

It has been shown recently that the whole CDI toxin (CdiA) can be found in the secreted fraction of the CDI producing strain (Roussin et al., 2019). Our results, using *A. baumannii* V15 strain with an up-regulated expression of *cdi* locus confirmed these observations (Figure 4A). We hypothesized that if CdiA is found in the supernatants of culture media, we should observe their toxicity against the capsule-negative *A. baumannii* strains. To test this, we supplied early logarithmic cultures of the capsule-negative *A. baumannii* strains (Δ*galU* mutants) II-c, II-a1, II-a (in a total volume of 150 μL) with 10 μL of 0.22 μm-filtered supernatant fractions of CDI^+^ and CDI^–^ strains and followed their growth. As can be seen in Figure 4B, the supernatant of CDI^+^ strain completely abolished the growth of the capsule negative II-a1 strain for at least 8 h, while the supernatant from the CDI^–^ strain had no impact. Consistently with the inter-bacterial competition assay, the capsule negative strain was rescued when it was complemented with either p*galU* or p*cdiI^V15^* plasmids, which restore capsule formation or supply immunity gene, respectively (Figure 4B). The same results were also obtained with the *A. baumannii* strains II-c and II-a (Supplementary Figures S1A,B), confirming that *A. baumannii* supernatants provide cell–cell contact independent toxicity.

It has been reported previously that fragments of CdiA protein are released from inhibitor cells but display no inhibitory activity (Aoki et al., 2005; Webb et al., 2013). Since we found that the supernatant of *A. baumannii* V15 strain displays toxicity against susceptible strains, we ought to test whether the effect is mediated by the secreted full size CdiA or only by the C-terminal toxic domain (CdiA-CT). CdiA is transported to the outside of the cell via CdiB, which is a membrane protein consisting of a β-barrel pore forming domain and two periplasmic polypeptide transport-associated (POTRA) domains, required for recognition and transport of the toxin (Fan et al., 2016) (Supplementary Figure S2). The CdiA is predicted to be 2133 amino acids long and ∼229 kDa in size (without the signal peptide). The CDI system can be assigned to type I CDI systems (De Gregorio et al., 2019). Our bioinformatic analysis showed that the protein contained a domain architecture, similar to that of already identified CdiA proteins. Its N-terminus contains a signal sequence required for transport across cytoplasmic membrane, followed by the domain, called two-partner secretion or filamentous hemagglutinin secretion, which is required for the transport via CdiB across the outer membrane. At the middle of the sequence it contains previously described heterogeneity (HET) region, predicted to participate in the target receptor binding (De Gregorio et al., 2019). Around this region we have identified two filamentous hemagglutinin repeat regions and two domains with the unknown function – DUF637 and DUF2345. Lastly, we found that C-terminus contained CdiA-CT region, which was demarcated by VENN motif (Supplementary Figure S2). Our analysis showed that CdiA-CT is 404 amino acids long or approximately 44 kDa in size (Supplementary Figure S2). It has been suggested that the organization of this region is similar among different CdiA toxins and is composed of N-terminal domain, which mediates transport through the inner-membrane into the target, while the C-terminal domain encodes the actual toxin (Willett et al., 2015a).

To evaluate whether the toxicity was caused by the full size protein or the CdiA-CT region, we have purified it from the culture supernatants of *A. baumannii* V15 Δ*bfmRS* strain as it displayed increased production of the CdiA. The cut-out band of the CdiA was also verified by mass spectrometry and results were consistent with the predicted CdiA protein as analysis detected only five filamentous hemagglutinins or large exoproteins with a predicted molecular weight of 200–228 kDa. The coverage of detected proteins ranged from 10 to 15% (Krasauskas et al., 2019). After using ammonium sulfate precipitation, hydrophobic interaction chromatography and protein concentration with molecular weight cut-off of 100 kDa, the CdiA protein with ∼70% purity was obtained (Figure 4C). We then performed size-exclusion chromatography of the purified CdiA, which revealed the approximate molecular weight of 152 kDa (Figure 4D), suggesting that the toxic component in the supernatant is only a part of the full size CdiA protein.

To confirm, that inhibition is protein-mediated, we also performed growth inhibition assays using purified CdiA fractions (0.3 μg/mL final concentration) that were pre-incubated with Proteinase K. This treatment abolished the inhibitory activity of purified fractions against all tested strains (Supplementary Figures S3A–C). We also determined that the minimum inhibitory concentration (MIC) of CdiA was 1.25 μg/mL for the Δ*galU* strains, while all other strains showed at least 10 times higher resistance (Supplementary Table S3). Taken together, the results allowed us to conclude that *A. baumannii* is able to secrete the large part of the CdiA protein into the culture media and that it is able to display inhibitory activity on the susceptible cells without a direct cell–cell contact.

### Secreted *A. baumannii* CdiA Protein Induces Cell Growth Arrest

**FIGURE 5:**
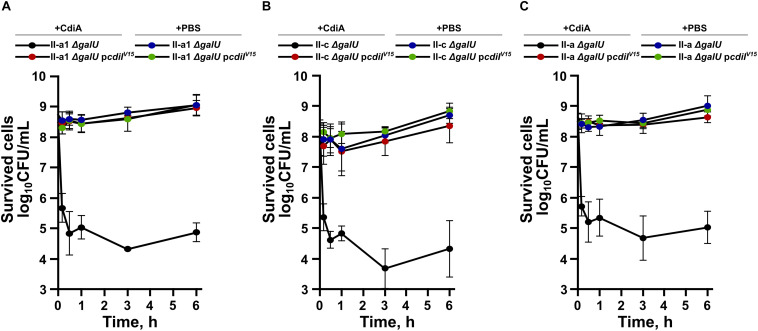
Time-dependent bacterial viability assays of *A. baumannii* clinical strains II-a1 **(A)**, II-c **(B)**, and II-a **(C)**, their *galU* mutants, and *galU* mutants complemented with immunity gene *cdiI*^V15^ from the *A. baumannii* V15. The viabilities after the addition of either PBS or the purified CdiA were assessed by measuring the remaining bacterial CFUs at the following time points: 0 h, 10 min, 30 min, 1 h, 3 h, and 6 h. Values were calculated from three independent experiments. Error bars represent standard deviation.

It has been determined, that while some CDI toxins cause cell death, others only arrest the growth of the susceptible strain (Aoki et al., 2009; Roussin et al., 2019). Therefore, we evaluated whether *A. baumannii* CdiA kills target cells by performing cell viability assay. The remaining cell count was evaluated by plating at different time points capsule-deficient and complemented with the immunity gene *A. baumannii* strains, mixed with the purified CdiA. In parallel, live/dead assay, using SYTO9 and propidium iodide stains, was applied. As can be seen in Figure 5A, a CdiA concentration of 4 × MIC on the 10^8^ CFU/mL of starting culture of strain II-a1, resulted in an immediate ∼500-fold reduction of the cell count after only 10 min. The cell count remained relatively unchanged for at least 6 h. The cell count of capsule-negative strains, complemented with immunity gene (p*cdiI*^V15^) or when the cells were mixed with PBS, remained unchanged (Figure 5A). Similar results were also obtained with the strains II-c and II-a (Figures 5B,C).

**FIGURE 6:**
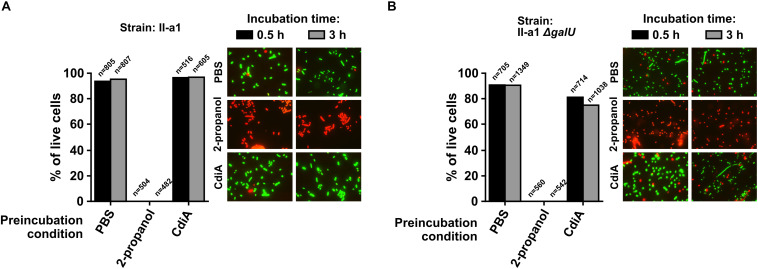
Live/dead assays performed with *A. baumannii* clinical strain II-a1 **(A)** and its *galU* mutant **(B)**. Graphs display the percentage of live cells. Bacteria, before each assay, were treated for 30 min or 3 h with either PBS (live control), 2-propanol (dead control), or the purified CdiA. Treated bacteria were visualized at 1,000× magnification with a fluorescence microscope Olympus AX70 equipped with 100×/1.35 oil immersion lens and WIBA (460–490 nm for excitation and 515–550 nm for emission) and MWG (510–550/590) filter cubes for SYTO9 and propidium iodide, respectively. Representative images of stained cells from each live/dead assay are provided next to graphs. Displayed data are from two separate biological replicates with similar outcomes. Numbers above columns indicate total number of bacterial cells counted.

To evaluate whether cells experienced death or growth inhibition, cell viability experiments were supported by the immediate live/dead staining of the II-a1 cells after pre-incubation of ∼10^8^ CFU/mL starting culture in PBS (live control), with 2-propanol (dead control) or with the purified CdiA protein for 30 min and for 3 h, in case cells death would occur later in time frame. Our results show that independent from incubation time with the CdiA protein, there were no apparent reduction in the numbers of live wild-type II-a1 cells as we observed approximately 90% of these cells (Figure 6A). The Δ*galU* mutant of II-a1 showed at least 75% of live cells when incubated with the CdiA for 30 min and 3 h (Figure 6B). Similar numbers of live cells were observed with the Δ*galU* mutant complemented with the p*cdiI^V15^* plasmid, while the complementation with the p*galU* caused indistinguishable phenotype from the wild-type strain (Supplementary Figures S4A,B). Therefore our data show that it is likely that CdiA from *A. baumannii* V15 causes target cell growth arrest.

## Discussion

In the present work, we have attempted to further elucidate contact-dependent growth inhibition mechanism of *A. baumannii*. We observed that capsule formation is essential for *A. baumannii* to be able to resist CDI-mediated inhibition. By using three capsule negative clinical *A. baumannii* strains and performing competition assays, we determined that they were significantly out-competed by strains expressing CDI system. This indicates that capsule might effectively hide a receptor or block CdiA-target cell contact, required for CDI-mediated inhibition. Capsule over-production has been previously implicated in the resistance to CDI-mediated inhibition in *E. coli* (Aoki et al., 2008). However, this phenotype has never been shown in *A. baumannii*. Our recent work has demonstrated that nearly all clinical *A. baumannii* isolates produce capsule (Skerniškytė et al., 2019), indicating that all of them might be resistant to CDI. Interestingly, there are reports, showing that capsule-deficient strains might display increased adherence to the surface due to increased hydrophobicity [(Kempf et al., 2012), also our unpublished data]. The increased adhesiveness could aid in the initial biofilm formation stages, where bacteria need to attach to the abiotic surface. In the case when the surface is already inhabited by other *A. baumannii* strain, adhesion of other capsule-deficient strains due to the susceptibility to CDI-mediated inhibition would be impossible, proving an effective *A. baumannii* defense strategy against population invaders. This phenotype could aid in the stabilization of genetically related populations and protect individuals within them from wasting resources on repeatedly delivering CDI toxins to the sister cells.

However, there have been observations that *A. baumannii* strains with thinner capsules do not display increased biofilm formation (Hu et al., 2020), suggesting that such modulation of capsule structure might be more favorable under other conditions. Recent research conducted using opaque and translucent colonies formed by the same strain due to phenotypic switching, identified two-fold difference in capsule thickness between them (Chin et al., 2018). It was suggested, that due to up-regulation of various metabolic and transport pathways, and increased biofilm formation at 25°C, the translucent cells are better suited for life in natural environments outside the host (Chin et al., 2018). The reduced capsule formation and at the same time increased susceptibility to CDI-mediated inhibition might be explained by the redirection of limited cell resources required for other cellular process, which are essential for the persistence in different environments.

Previous work has shown that *A. baumannii* two-component system BfmRS reduces capsule production in some strains but not others (Geisinger and Isberg, 2015; Russo et al., 2016). Our results indicate that BfmRS deletion did not lead to an apparent reduction in the quantity of the capsule, and are in agreement with the previously published results with *A. baumannii* AB307-0294 strain (Russo et al., 2016). However, we observed that *A. baumannii*Δ*bfmRS* mutants displayed a different capsular polysaccharide profiles in terms of polymers with a more wider range of molecular weight. This phenotype might have been caused by the inability of mutants to polymerize the correct size of capsular polysaccharide. It has been observed that in Gram-negative bacteria with Group 1 capsular polysaccharides, the protein Wzc is responsible for the regulation of synthesis and assembly of high-molecular-weight polymers (Whitfield, 2006). Consistently, a recent study demonstrated that *A. baumannii* Wzc protein controls capsule polymer chain length depending on the auto-phosphorylation level (Geisinger and Isberg, 2015). A follow-up study identified that the *bfmRS* deletion causes an overall down-regulation of the capsule synthesis locus (Geisinger et al., 2018). The disproportionate or reduced level of proteins required for capsular polysaccharide synthesis might impact the ability of *A. baumannii* to efficiently synthesize the correct chain length polymer.

Interestingly, competition assays showed that at least one clinical isolate with Δ*bfmRS* deletion became susceptible to CDI-mediated inhibition indicating that the BfmRS system in some strains, via yet unknown mechanism, may determine the susceptibility against CDI. Such strain-depended behavior could be a consequence of a variation of surface-related features of the certain strain that were described in the previous study (Skerniškytė et al., 2019). Although, the strains expressed diverse phenotypes in cell surface hydrophobicity, biofilm formation and adherence to epithelium cells *in vitro*, there were no correlation between these features and capsule or lipooligosaccharide synthesis genetic locus type. A recent publication described that *E. coli* and *Vibrio cholerae* can mount an immunity-independent resistance against type VI-secretion system via envelope stress response modulators – Rcs phosphorelay (*E. coli*) and two-component systems BaeSR (*E. coli*) and WigKR (*V. cholerae*). Authors showed that responses include such phenotypes as up-regulation of osmotic-response genes, increased capsular polysaccharide synthesis, and general cell integrity preservation (Hersch et al., 2020). It is tempting to speculate, that BfmRS system might similarly cause immunity-independent resistance phenotype against CDI as in addition to the role in capsule formation in some *A. baumannii* strains, it also contributes to resistance to high osmolarity and desiccation (Farrow et al., 2018). However, these are complex phenotypes regulated by multiple factors, therefore it is possible that in some strains the loss of *bfmRS* might be compensated by other regulatory pathways.

It has been long known that *A. baumannii* BfmRS system is required for biofilm formation on the abiotic surfaces (Tomaras et al., 2008). Biofilm formation phenotype effectively protects the colony from various unfavorable environment conditions such as desiccation, chemical perturbations, competition from other bacteria, killing by predators, or presence of antibiotics (Yan and Bassler, 2019). However, it must be noted that formation of biofilm requires synthesis and secretion of various adhesion factors, nutrient assimilation factors, exopolysaccharides, lipopolysaccharide, and others (Flemming and Wingender, 2010). These factors can be viewed as resources, which, after secretion by bacteria, become available to other cells within a population, potentially allowing to be exploited by strains that do not produce them (Xavier, 2011). The fact that we observed at least one strain to be directly susceptible to CDI-mediated inhibition due to the loss of *bfmRS* operon, suggests that the biofilm formation circuitry in some *A. baumannii* strains has a CDI-dependent policing mechanism to those population individuals that do not contribute to biofilm formation. The policing strategies against non-cooperative strains in a population were reported in other bacterial pathogens such as *Pseudomonas aeruginosa* (Wang et al., 2015). However, our results also indicate that BfmRS system might play a strain-specific role in *A. baumannii* as we observed that this phenotype was not universal among tested isolates.

In this work, we also provide evidence that *A. baumannii* CdiA protein can be found in the growth media and despite being detached from the cell, still efficiently induces growth arrest of a susceptible strain without the need for inter-cellular contact. The phenomenon of CDI was first described in a wild-type *E. coli* EC93 isolate, which inhibited growth of other strains in the co-culture (Aoki et al., 2005). Despite the fact that fragments of CdiA were found in the growth media, they were seemingly inactive, therefore it was concluded that inhibition requires a direct cell–cell contact (Aoki et al., 2005), hence the term CDI was coined. It has since been well documented that CDI, along with Type VI secretion system (T6SS) comprise a group of cell–cell contact requiring mechanisms for delivering effectors to the neighboring cells (Danka et al., 2017). Our results indicate, that *A. baumannii* is able to secrete or shed from the outer membrane a fully functional CdiA protein into the growth media, thereby expanding the area of influence. It is thought that after export, CdiA remains non-covalently bound to CdiB on the outer membrane (Willett et al., 2015b), therefore shedding into the growth media could occur naturally. However, the question remains whether it is an active mechanism and primary objective of CdiA or is a side-effect. Recent work by Bottery et al. (2019), using combination of computational and synthetic biology approaches has shown, that CDI systems are used to generate mixed-strain populations by creating single-cell-wide boundaries between them. The ability to generate the soluble CdiA effector could induce a reductive shift of population genetic heterogeneity thereby preventing such boundaries and allowing the establishment of a population of only related bacteria. On the other hand, a study conducted by Allen et al. (2020) revealed that a functional CDI system from *P. aeruginosa* not only participates in inter-bacterial competition but also plays a role in the virulence in mouse infection model and exhibits cytotoxicity against eukaryotic cells. It is tempting to speculate that a similar mechanism could be utilized by *A. baumannii* to more effectively colonize its host. However, it must be noted that CdiA in *A. baumannii* V15 is up-regulated by the loss of *bfmRS* (Krasauskas et al., 2019). It is well known that the loss of *bfmRS* causes various fitness defects (Wang et al., 2014; Gebhardt et al., 2015; Geisinger and Isberg, 2015; Russo et al., 2016; Farrow et al., 2018; Geisinger et al., 2018), which would prevent the effective colonization of the host. Therefore, a direct CDI participation in *A. baumannii* virulence is unlikely.

In this study, we also showed that *A. baumannii* CdiA protein causes a growth arrest of a susceptible bacteria. Our homology search using HHPRED (Zimmermann et al., 2018) revealed a weak sequence similarity of CdiA-CT to known proteins, however, a distant homology to toxin-deaminases, belonging to one of the most common toxin domains (Zhang et al., 2012) was observed. The most similar hit (84% probability) was found to be to a part of human APOBEC-2 deaminase, involved in deamination of cytidine in mRNA and single-stranded DNA (Prochnow et al., 2007). Unfortunately, we were unable to specify CdiA functional activity in the purified fraction. The proposed mechanism of CDI inhibition suggests that prior target binding, the secretion full CdiA protein is inhibited, the N-terminal-half filament of the protein localizing outside the cell, while C-terminal-half positioning in the periplasm. After binding the target receptor via the receptor binding domain on the distal tip of the N-terminal half, the C-terminal half of CdiA is translocated out of the periplasm and integrated into the outer membrane of the target cell, leading to a subsequent translocation of CdiA-CT into the periplasm of the target. There, CdiA-CT is cleaved and translocated into the cytoplasm (Ruhe et al., 2018). We hypothesize that the secreted CdiA protein upon binding its receptor remains inactive, preventing the activity measurements.

In sum, our work indicates, that capsule protects *A. baumannii* from CDI-mediated competition within species. Moreover, we demonstrate that BfmRS system could also impact resistance of some strains against CDI, suggesting that several mechanisms are in place, which protect *A. baumannii* from CDI. Finally, for the first time we were able to show, that *A. baumannii* effector protein CdiA can be secreted into the growth media and retains functional activity by causing the growth arrest of the susceptible *A. baumannii* cells, thereby indicating that cell contact might not be necessary for CDI system in *A. baumannii* V15.

## Data Availability Statement

All datasets generated for this study are included in the article/Supplementary Material.

## Author Contributions

RK and ES conceived and designed the experiments, analyzed the data, and wrote the manuscript. RK, JS, JM, and JA performed the experiments. All authors read and approved the final manuscript.

## Conflict of Interest

The authors declare that the research was conducted in the absence of any commercial or financial relationships that could be construed as a potential conflict of interest.
