# Erratum: Capsule Protects *Acinetobacter baumannii* From Inter-Bacterial Competition Mediated by CdiA Toxin

**DOI:** 10.3389/fmicb.2020.598762

**Published:** 2020-09-15

**Authors:** Frontiers Production Office

**Affiliations:** Frontiers Media SA, Lausanne, Switzerland

**Keywords:** *Acinetobacter baumannii*, CdiA, capsule, BfmRS, contact-dependent inhibition

Due to a production error, several references with more than six authors did not include “et al.” The publisher apologizes for this mistake.

The original article has been updated.

