# Supplementary material for: Capsule Protects Acinetobacter baumannii From Inter-Bacterial Competition Mediated by CdiA Toxin
Source: Front Microbiol. 2020 Jul 17;11:1493. doi: 10.3389/fmicb.2020.01493 (PMC7396552; doi:10.3389/fmicb.2020.01493)
Supplement: Supplementary file 2 [file Image_2.pdf]

Supplementary Material

Capsule Protects *Acinetobacter baumannii* From Inter-Bacterial Competition Mediated by CdiA Toxin

Renatas Krasauskas[1*], Jūratė Skerniškytė[1], Julius Martinkus[1], Julija Armalytė[1], Edita Sužiedėlienė[1]

[1]Institute of Biosciences, Life Sciences Center, Vilnius University, Vilnius, Lithuania

*** Correspondence:**
Renatas Krasauskas
renatas.krasauskas@gf.vu.lt

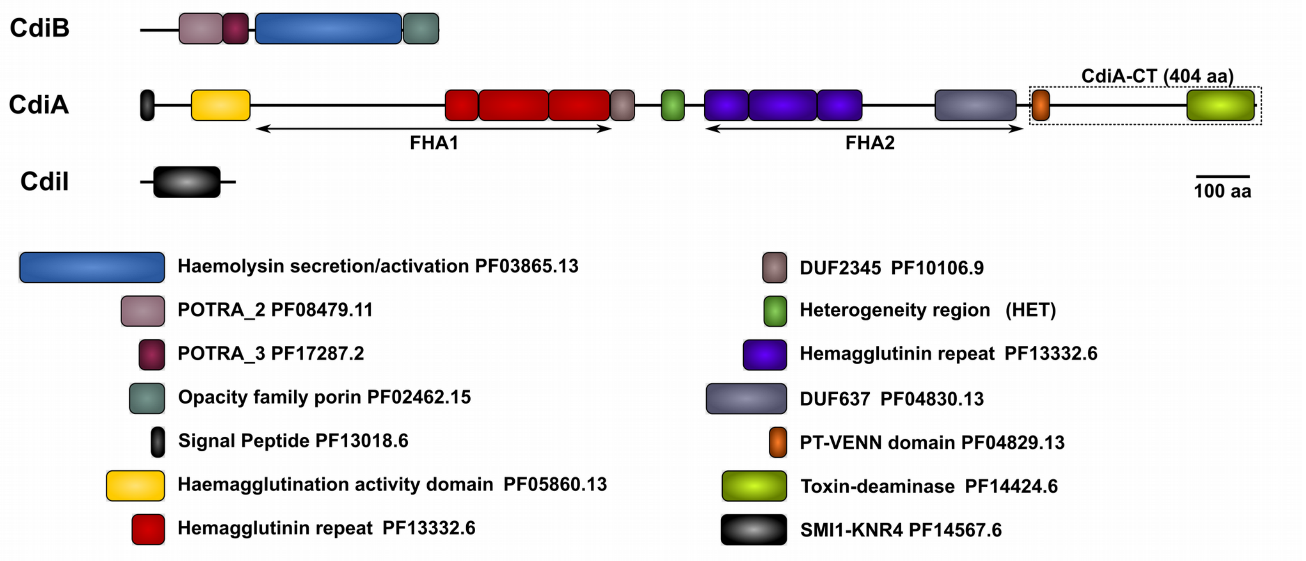

Supplementary Figure S2. Domain organisation of proteins encoded on the *cdiBAI* locus from *Acinetobacter baumannii* V15 (Genbank accession code: MK405474.1). Pfam database accession codes are provided next to the names of the domains identified. Predictions were performed using JACKHMMER (HmmerWeb version 2.41.1) by iterative search (up to 4 iterations) with sequences from the whole and/or part of the protein of interest against rp55 database (version 2020_01) with default parameters. Alignments with the resulting hits were then used as queries to perform searches using HHPRED web version (Zimmermann *et al.* 2018) against Pfam 32.0 database. Hits were determined as significant if probability value was above 80 %. The proposed HET region (De Gregorio *et. al.* 2019) was identified by MARCOIL program. Please note that the two-partner secretion domain here is represented by haemagglutination activity domain.

Supplementary Material

References.

De Gregorio, E., Zarrilli, R., and Di Nocera, P. P. (2019). Contact-dependent growth inhibition systems in Acinetobacter. *Sci Rep* 9, 154. doi:10.1038/s41598-018-36427-8.

Zimmermann, L., Stephens, A., Nam, S.-Z., Rau, D., Kübler, J., Lozajic, M., et al. (2018). A Completely Reimplemented MPI Bioinformatics Toolkit with a New HHpred Server at its Core. *J. Mol. Biol.* 430, 2237–2243. doi:10.1016/j.jmb.2017.12.007.
