# Supplementary material for: Capsule Protects Acinetobacter baumannii From Inter-Bacterial Competition Mediated by CdiA Toxin
Source: Front Microbiol. 2020 Jul 17;11:1493. doi: 10.3389/fmicb.2020.01493 (PMC7396552; doi:10.3389/fmicb.2020.01493)
Supplement: Supplementary file 3 [file Image_3.PDF]

frontiers

Supplementary Material

Capsule Protects *Acinetobacter baumannii* From Inter-Bacterial Competition Mediated by CdiA Toxin

Renatas Krasauskas[1*], Jūratė Skerniškytė[1], Julius Martinkus[1], Julija Armalytė[1], Edita Sužiedėlienė[1]

[1]Institute of Biosciences, Life Sciences Center, Vilnius University, Vilnius, Lithuania

*** Correspondence:**
Renatas Krasauskas
renatas.krasauskas@gf.vu.lt

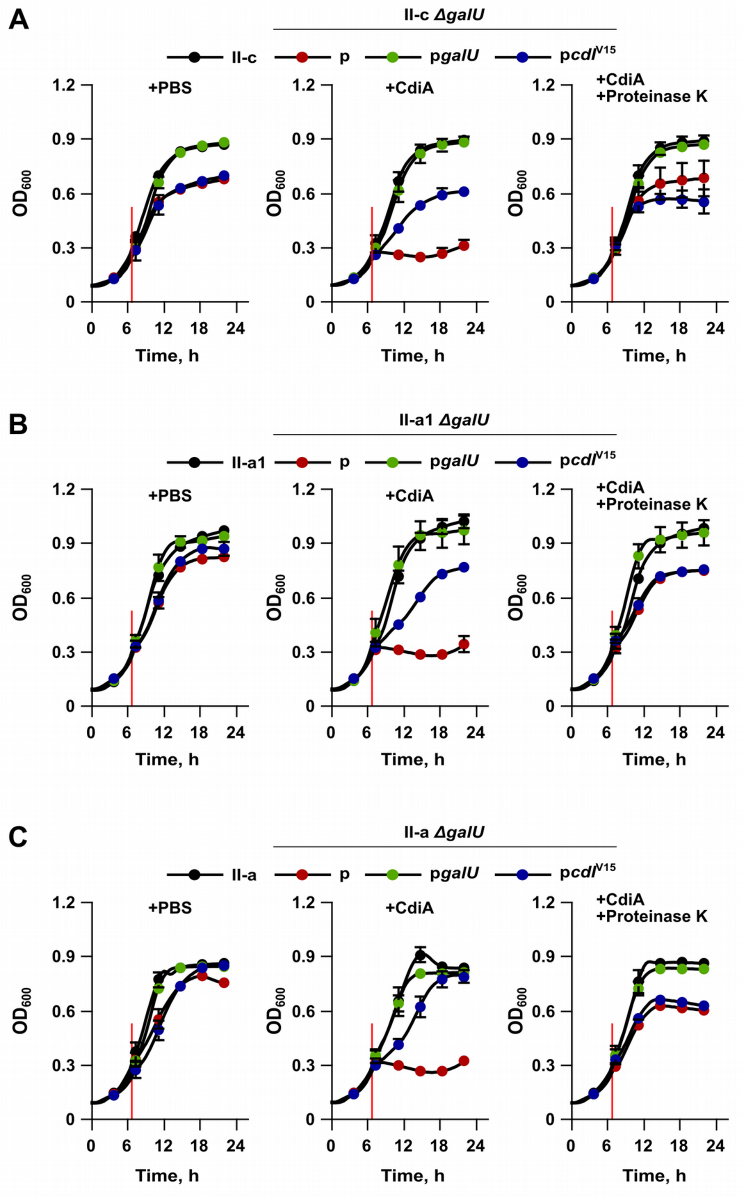

Supplementary Figure S3. (**A-C**) Growth inhibition assays of *A. baumannii* clinical strains II-c (**A**), II-a1 (**B**), II-a (**C**) their *galU* mutants, and *galU* mutants complemented with either wild-type *galU* gene or immunity gene *cdiI*^{V15} from the *A. baumannii* V15 strain. Red vertical lines denote the time when 10 μL of either PBS, 50 ng of purified CdiA, or 50 ng of purified CdiA pre-incubated with Proteinase K for 30 min were added. Values were calculated from three independent experiments each with two technical replicates. Error bars represent standard deviation.
