# Supplementary material for: Capsule Protects Acinetobacter baumannii From Inter-Bacterial Competition Mediated by CdiA Toxin
Source: Front Microbiol. 2020 Jul 17;11:1493. doi: 10.3389/fmicb.2020.01493 (PMC7396552; doi:10.3389/fmicb.2020.01493)
Supplement: Supplementary file 4 [file Image_4.PDF]

Supplementary Material

Capsule Protects *Acinetobacter baumannii* From Inter-Bacterial Competition Mediated by CdiA Toxin

Renatas Krasauskas[1*], Jūratė Skerniškytė[1], Julius Martinkus[1], Julija Armalytė[1], Edita Sužiedėlienė[1]

[1]Institute of Biosciences, Life Sciences Center, Vilnius University, Vilnius, Lithuania

*** Correspondence:**
Renatas Krasauskas
renatas.krasauskas@gf.vu.lt

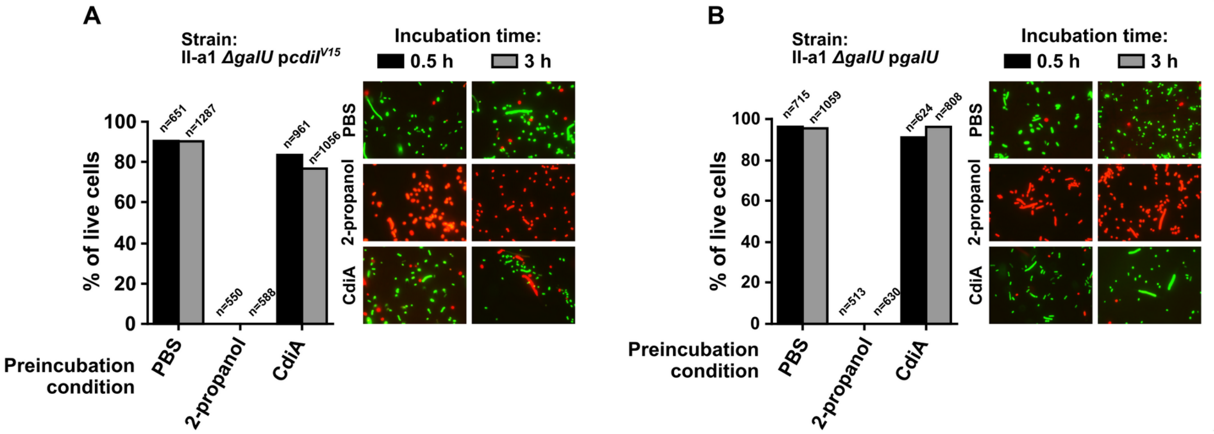

Supplementary Figure S4. Live/dead assays performed with the *galU* mutant of *A. baumannii* clinical strain II-a1, complemented with plasmids p*cdiI*^{V15} (A) and p*galU* (B), encoding either immunity gene from the *A. baumannii* V15 strain or wild-type *galU* gene, respectively. Graphs display the percentage of live cells. Bacteria before each assay were treated for 30 minutes or 3 hours with either PBS (live control), 2-propanol (dead control), or the purified CdiA. Treated bacteria were visualized at 1000x magnification with a fluorescence microscope Olympus AX70 equipped with 100x/1.35 oil immersion lens and WIBA (460–490 nm for excitation and 515–550 nm for emission) and MWG (510-550/590) filter cubes for SYTO9 and propidium iodide, respectively. Representative images of stained cells from each live/dead assay are provided next to graphs. Displayed data are from two separate biological replicates with similar outcomes. Numbers above columns indicate total number of bacterial cells counted.
