# Supplementary material for: Capsule Protects Acinetobacter baumannii From Inter-Bacterial Competition Mediated by CdiA Toxin
Source: Front Microbiol. 2020 Jul 17;11:1493. doi: 10.3389/fmicb.2020.01493 (PMC7396552; doi:10.3389/fmicb.2020.01493)
Supplement: Supplementary file 5 [file Table_1.doc]

Supplementary Material

Capsule Protects *Acinetobacter baumannii* From Inter-Bacterial Competition Mediated by CdiA Toxin

Renatas Krasauskas^1*^, Jūratė Skerniškytė^1^, Julius Martinkus^1^, Julija Armalytė^1^, Edita Sužiedėlienė^1^

^1^Institute of Biosciences, Life Sciences Center, Vilnius University, Vilnius, Lithuania

*** Correspondence:**Renatas Krasauskas
[renatas.krasauskas@gf.vu.lt](mailto:renatas.krasauskas@gf.vu.lt)

**Supplementary Table S1.** Bacterial strains and plasmids used in the study.

| **Strain or plasmid** | **Description and/or genotype** | **Reference** |
| --- | --- | --- |
| ***Escherichia coli*** | | |
| JM107 | General laboratory strain used for all cloning experiments and DNA manipulations.  *endA1*, *glnV44*, *thi*^-^, *relA1*, *gyrA96*, *Δ(lac-proAB)* [F', *traD36*, *proAB*^+^, *lacI^q^ZΔM15*], *hsdR17*(R_K_^-^ m_K_^+^), λ^-^. | (Yanisch-Perron, Vieira, and Messing 1985) |
| DH5α | Strain used for inter-bacterial competitive growth assays as a negative non-competitive control.  F^-^, Δ*(argF-lac)*169, *φ80dlacZ58(M15)*, *ΔphoA8*, *glnX44(AS)*, *λ^-^*, *deoR481*, *rfbC1*, *gyrA96(NalR)*, *recA1*, *endA1*, *thiE1*, *hsdR17*. | (Woodcock *et al.* 1989) |
| ***Acinetobacter baumannii*** | | |
| V15 | Clinical isolate, IC_other, unique pulsotype U18, ST1422. | (Povilonis *et al.* 2013) |
| V15 *ΔbfmRS* | V15 derivative with deleted *bfmRS* operon. | (Krasauskas *et al.* 2019) |
| V15 *Δhcp* | V15 derivative with deleted *hcp* gene. |
| V15 *ΔbfmRSΔhcp* | V15 *ΔbfmRS* derivative with deleted *hcp* gene. |
| V15 *ΔhcpΔcdi^V15^* | V15 *Δhcp* derivative with partial deletion of *cdiBAI^V15^* operon. |
| V15 *ΔbfmRSΔcdi^V15^* | V15 *ΔbfmRS* derivative with partial deletion of *cdiBAI^V15^* operon. |
| V15 *ΔbfmRSΔhcpΔcdi^V15^* | V15 *ΔbfmRSΔhcp* derivative with partial deletion of *cdiBAI^V15^* operon. |
| II-a1 | Clinical *A. baumannii* isolate, IC II, pulsotype II-a1, ST2. | (Povilonis *et al.* 2013; Skerniškytė *et al.* 2019) |
| II-a | Clinical *A. baumannii* isolate, IC II, pulsotype II-a, ST2. |
| II-c | Clinical *A. baumannii isolate*, IC II, pulsotype II-c, ST2. |
| II-a1 *ΔgalU* | II-a1 with deleted *galU* gene. | This study |
| II-a *ΔgalU* | II-a with deleted *galU* gene. | This study |
| II-c *ΔgalU* | II-c with deleted *galU* gene. | This study |
| II-a1 *ΔgalU* p*galU* | II-a1 *ΔgalU* complemented with pUC_AcORI_Ptac_galU^II-a1^_TER_lacIq2 | This study |
| II-a *ΔgalU* p*galU* | II-a *ΔgalU* complemented with pUC_AcORI_Ptac_galU^II-a^ _TER_lacIq2 | This study |
| II-c *ΔgalU* p*galU* | II-c *ΔgalU* complemented with pUC_AcORI_Ptac_galU^II-c^ _TER_lacIq2 | This study |
| II-a1 *ΔbfmRS* | II-a1 with deleted *ΔbfmRS* operon. | This study |
| II-a *ΔbfmRS* | II-a with deleted *ΔbfmRS* operon. | This study |
| II-c *ΔbfmRS* | II-c with deleted *ΔbfmRS* operon. | This study |
| II-a1 *ΔbfmRS* p*bfmRS* | II-a1 *ΔbfmRS* complemented with pUC_AcORI_Ptac_*bfmRS*^V15^_TER_lacIq2 | This study |
| II-a *ΔbfmRS* p*bfmRS* | II-a *ΔbfmRS* complemented with pUC_AcORI_Ptac_*bfmRS*^V15^_TER_lacIq2 | This study |
| II-c *ΔbfmRS* p*bfmRS* | II-c *ΔbfmRS* complemented with pUC_AcORI_Ptac_*bfmRS*^V15^_TER_lacIq2 | This study |
|  |  |  |
| **Plasmids** | | |
| pUC19_sacB | *A. baumannii* suicide vector containing *sacB* gene from *Bacillus* spp. cloned via *Xba*I and *Pae*I; Amp^R^. | (Krasauskas *et al.* 2019) |
| pUC19_sacB_galU^II-a1^UPDwn_gmR | pUC19_sacB derivative with *ΔgalU*^II-a1^::aac3I; Amp^R^; Gm^R^. | This study |
| pUC19_sacB_galU^II-a^UPDwn_gmR | pUC19_sacB derivative with *ΔgalU*^II-a^::aac3I; Amp^R^; Gm^R^. | This study |
| pUC19_sacB_galU^II-c^UPDwn_gmR | pUC19_sacB derivative with *ΔgalU*^II-c^::aac3I; Amp^R^; Gm^R^. | This study |
| pUC19_sacB_bfmRS^II-a1^UPDwn_gmR | pUC19_sacB derivative with *ΔbfmRS*^II-a1^::aac3I; Amp^R^; Gm^R^. | This study |
| pUC19_sacB_bfmRS^IIa^UPDwn_gmR | pUC19_sacB derivative with *ΔbfmRS*^II-a^::*aac3I*; Amp^R^; Gm^R^. | This study |
| pUC19_sacB_bfmRS^IIc^UPDwn_gmR | pUC19_sacB derivative with *ΔbfmRS*^II-c^::*aac3I*; Amp^R^; Gm^R^. | This study |
| pUC_AcORI_Ptac_gfp_TER_lacIq2 | Leaky/inducible *A. baumannii* plasmid with P*_tac_* promoter; Amp^R^. | (Krasauskas *et al.* 2019) |
| pUC_AcORI_Ptac_gfp_TER_lacIq2_gm | pUC_AcORI_Ptac_gfp_TER_lacIq2 derivative, where *bla* is replaced with *aac3I*; Gm^R^. | This study |
| pUC_AcORI_Ptac_galU^II-a1^_TER_lacIq2_gm | pUC_AcORI_Ptac_gfp_TER_lacIq2 derivative, where *gfp* gene is replaced with a wild-type *galU* gene from II-a1; Gm^R^. | This study |
| pUC_AcORI_Ptac_galU^II-a^_TER_lacIq2_gm | pUC_AcORI_Ptac_gfp_TER_lacIq2 derivative, where *gfp* gene is replaced with a wild-type *galU* gene from II-a; Gm^R^. | This study |
| pUC_AcORI_Ptac_galU^II-c^_TER_lacIq2_gm | pUC_AcORI_Ptac_gfp_TER_lacIq2 derivative, where *gfp* gene is replaced with a wild-type *galU* gene from II-c; Gm^R^. | This study |
| pUC_AcORI_Ptac_bfmRS^V15^_TER_lacIq2 | pUC_AcORI_Ptac_gfp_TER_lacIq2 derivative ,where *gfp* gene is replaced with *bfmRS* operon from V15; Amp^R^. | (Krasauskas *et al.* 2019) |
| pUC_AcORI_Ptac_bfmRS^V15^_TER_lacIq2_gm | pUC_AcORI_Ptac_bfmRS^V15^_TER_lacIq2 derivative, where *bla* is replaced with *aac3I*; Gm^R^. | This study |
| pUC_AcORI_Ptac_cdiI^V15^_TER_lacIq2_gm | pUC_AcORI_Ptac_gfp_TER_lacIq2 derivative, where *gfp* is replaced with *cdiI*^V15^, and *bla* is replaced with *aac3I*; Gm^R^. | (Krasauskas *et al.* 2019) |
|  |  |  |

Amp^R^, ampicillin resistant; Gm^R^, gentamicin resistant;

# References

Krasauskas, R., Skerniškytė, J., Armalytė, J., and Sužiedėlienė, E. (2019). The role of *Acinetobacter* *baumannii* response regulator BfmR in pellicle formation and competitiveness via contact-dependent inhibition system. *BMC Microbiol.* 19, 241. doi:[10.1186/s12866-019-1621-5](https://doi.org/10.1186/s12866-019-1621-5).

Povilonis, J., Seputiene, V., Krasauskas, R., Juskaite, R., Miskinyte, M., Suziedelis, K., et al. (2013). Spread of carbapenem-resistant *Acinetobacter* *baumannii* carrying a plasmid with two genes encoding OXA-72 carbapenemase in Lithuanian hospitals. *J. Antimicrob. Chemother.* 68, 1000–1006. doi:[10.1093/jac/dks499](https://doi.org/10.1093/jac/dks499).

Skerniškytė, J., Krasauskas, R., Péchoux, C., Kulakauskas, S., Armalytė, J., and Sužiedėlienė, E. (2019). Surface-Related Features and Virulence Among *Acinetobacter* *baumannii* Clinical Isolates Belonging to International Clones I and II. *Front Microbiol* 9, 3116. doi:[10.3389/fmicb.2018.03116](https://doi.org/10.3389/fmicb.2018.03116).

Woodcock, D. M., Crowther, P. J., Doherty, J., Jefferson, S., DeCruz, E., Noyer-Weidner, M., et al. (1989). Quantitative evaluation of *Escherichia* *coli* host strains for tolerance to cytosine methylation in plasmid and phage recombinants. *Nucleic Acids Res.* 17, 3469–3478. doi:[10.1093/nar/17.9.3469](https://doi.org/10.1093/nar/17.9.3469).

Yanisch-Perron, C., Vieira, J., and Messing, J. (1985). Improved M13 phage cloning vectors and host strains: nucleotide sequences of the M13mp18 and pUC19 vectors. *Gene* 33, 103–119. doi:[10.1016/0378-1119(85)90120-9](https://doi.org/10.1016/0378-1119(85)90120-9).
