# Supplementary material for: Capsule Protects Acinetobacter baumannii From Inter-Bacterial Competition Mediated by CdiA Toxin
Source: Front Microbiol. 2020 Jul 17;11:1493. doi: 10.3389/fmicb.2020.01493 (PMC7396552; doi:10.3389/fmicb.2020.01493)
Supplement: Supplementary file 6 [file Table_2.doc]

Supplementary Material

Capsule Protects *Acinetobacter baumannii* From Inter-Bacterial Competition Mediated by CdiA Toxin

Renatas Krasauskas^1*^, Jūratė Skerniškytė^1^, Julius Martinkus^1^, Julija Armalytė^1^, Edita Sužiedėlienė^1^

^1^Institute of Biosciences, Life Sciences Center, Vilnius University, Vilnius, Lithuania

*** Correspondence:**Renatas Krasauskas
[renatas.krasauskas@gf.vu.lt](mailto:renatas.krasauskas@gf.vu.lt)

**Supplementary Table S2**. Primers used in the study.

| **Oligonucleotide** | **Oligonucleotide sequence (5’→3’)** | **Description** |
| --- | --- | --- |
| galu_Up_F | GTCCGTAAAAATTTAGGTTC | Amplification of a *galU* upstream region. Overlay with *galU* downstream region is underlined. |
| galu_Up_R | CAAATAGTTAAGCAGAGCTACGTAGAACTGCTTTTTTAATCAT |
| galu_Dwn_F | GTAGCTCTGCTTAACTATTTG | Amplification of a *galU* downstream region. Overlay with *aac3I* downstream region is underlined. |
| galu_Dwn_R_gmR | CGTTCAAGCCGAGATGAATTCGATCCGGAATAAAATTTCTTTTTGTTG |
| galU_compl_F | cATGATTAAAAAGGCAGTTTTACCT | Amplification of a *galU*. Cloning into inducible vector. |
| galU_compl_R | CAAATACTTAAGCAGAGCTAC |
| galu_check | CTGCTTCCATGCCGTAACTA | Verification for a *galU* deletion. |
| galu_check2 | TTGTTTCCATGCGGTTACTA |
| BfmR_F | GTTGAAGCTTAAATGCAGCAACATCTCC | Verification for the *ΔbfmRS* deletion. Sequence obtained from (Tomaras et al., 2008). |
| BfmR01F | TCACGCATTGCACCATAA | Amplification of *bfmRS* upstream region. Overlay with *bfmRS* downstream region is underlined |
| BfmRS01R | GGAACCTGATGCAACTCAGTTATAAATCATTGCCCCTATAAATCTC |
| BfmS02F | TTATAACTGAGTTGCATCAGG | Amplification of *bfmRS* downstream region. Overlay with *aac3I* downstream region is underlined |
| BfmS02Rgm | CGTTCAAGCCGAGATGAATTCGATCGGCCGAATTTGGTTATTG |
| GentR_F | GATCGAGCTCAGGACAGAAATGCCTCGACT | Amplification of *aac3I* gene *Sac*I and *Eco*RI restriction sites are underlined |
| GentR_R | GATCGAATTCATCTCGGCTTGAACGAATTG |
| Bfm_check_F | CAACACCCTGAGATTTACCG | Verification for the *ΔbfmRS* deletion |
| Bfm_check_R | CAGCAACTTTTGTGCCTATG |
| Cdi_Imm_F | TTAAAGTAAACGACCTGTAATAGACC | Amplification of *cdiI^V15^*. Check of the presence of *cdiI*^V15^ |
| Cdi_Imm_R | CATGATCGATTTTGTTAAAGAATTATCTGC |

**References**

Tomaras, A. P., Flagler, M. J., Dorsey, C. W., Gaddy, J. A., and Actis, L. A. (2008). Characterization of a two-component regulatory system from Acinetobacter baumannii that controls biofilm formation and cellular morphology. *Microbiology (Reading, Engl.)* 154, 3398–3409. doi:[10.1099/mic.0.2008/019471-0](https://doi.org/10.1099/mic.0.2008/019471-0).
