# Supplementary material for: Capsule Protects Acinetobacter baumannii From Inter-Bacterial Competition Mediated by CdiA Toxin
Source: Front Microbiol. 2020 Jul 17;11:1493. doi: 10.3389/fmicb.2020.01493 (PMC7396552; doi:10.3389/fmicb.2020.01493)
Supplement: Supplementary file 7 [file Table_3.doc]

Supplementary Material

Capsule Protects *Acinetobacter baumannii* From Inter-Bacterial Competition Mediated by CdiA Toxin

Renatas Krasauskas^1*^, Jūratė Skerniškytė^1^, Julius Martinkus^1^, Julija Armalytė^1^, Edita Sužiedėlienė^1^

^1^Institute of Biosciences, Life Sciences Center, Vilnius University, Vilnius, Lithuania

*** Correspondence:**Renatas Krasauskas
[renatas.krasauskas@gf.vu.lt](mailto:renatas.krasauskas@gf.vu.lt)

**Supplementary Table S3.**  Minimal inhibitory concentration (MIC) values (μg/mL) of the clinical *A. baumannii* strains used in the work and their derivatives for the purified CdiA protein. The MIC values were determined by the broth micro-dilution method with LB media as described in (Wiegand et al., 2008).

| **Strain** | **MIC value, μg/mL** |
| --- | --- |
| II-a | >15 |
| II-a *ΔgalU* | 1.25 |
| II-a *ΔgalU* + p*galU* | >15 |
| II-a *ΔgalU* + p*cdiI*^V15^ | >15 |
| II-a1 | >15 |
| II-a1 *ΔgalU* | 1.25 |
| II-a1 *ΔgalU* + p*galU* | >15 |
| II-a1 *ΔgalU* + p*cdiI*^V15^ | >15 |
| II-c | >15 |
| II-c *ΔgalU* | 1.25 |
| II-c *ΔgalU* + p*galU* | >15 |
| II-c *ΔgalU* + p*cdiI*^V15^ | >15 |

**References.**

Wiegand, I., Hilpert, K., and Hancock, R. E. W. (2008). Agar and broth dilution methods to determine the minimal inhibitory concentration (MIC) of antimicrobial substances. *Nat Protoc* 3, 163–175. doi:[10.1038/nprot.2007.521](https://doi.org/10.1038/nprot.2007.521).
